# Kaposi’s varicelliform eruption in a patient with metastatic melanoma and primary cutaneous anaplastic large cell lymphoma treated with talimogene laherparepvec and nivolumab

**DOI:** 10.1186/s40425-018-0437-4

**Published:** 2018-11-19

**Authors:** David M. Miller, Ryan M. Trowbridge, Anupam Desai, Reed E. Drews

**Affiliations:** 1 Division of Hematology/Oncology, Beth Israel Deaconess Medical Center, Boston, MA USA; 2 Department of Dermatology, Massachusetts General Hospital, 50 Staniford Street, Boston, MA 02114 USA; 3 Department of Dermatology, University of Nebraska Medical Center, Omaha, NE USA

**Keywords:** Talimogene laherparepvec, Melanoma, Anaplastic large cell lymphoma, Kaposi varicelliform eruption, eczema TVECium, Immunocompromised

## Abstract

**Background:**

Immune-directed therapies have become front-line therapy for melanoma and are transforming the management of advanced disease. In refractory cases, multi-modal immunoncology (IO) approaches are being utilized, including combining immune checkpoint blockade (ICB) with oncolytic herpes viruses. Talimogene laherparepvec (T-VEC) is the first genetically modified oncolytic viral therapy (OVT) approved for the treatment of recurrent and unresectable melanoma. The use of IO in patients with concomitant malignancies and/or compromised immune systems is limited due to systematic exclusion from clinical trials. For example, a single case report of a solid organ transplant patient successfully treated with T-VEC for metastatic melanoma has been reported. Furthermore, the use of ICB in T-cell malignancies is limited and paradoxical worsening has been described. To our knowledge, this is the first report of dual ICB/T-VEC being administered to a patient with concurrent primary cutaneous anaplastic large cell lymphoma (pcALCL) and melanoma.

**Case presentation:**

Here we present the case of a patient with concomitant primary cutaneous ALCL and metastatic melanoma, progressing on anti-programmed death (PD)-1 therapy, who developed Kaposi’s varicelliform eruption after receiving the first dose of Talimogene laherparepvec.

**Conclusion:**

This case highlights the complexities of care of patients with coexistent cancers, demonstrates rapid progression of primary cutaneous ALCL on nivolumab and introduces a novel adverse effect of Talimogene laherparepvec.

## Background

Immune-directed therapies have become front-line for the vast majority of advanced melanoma cases. ICB with anti-PD-1 antibodies are routinely used as first-line options for patients with high-risk resected or metastatic disease. For patients with unresectable disease, oncolytic virus therapy with talimogene laherparepvec (T-VEC, or Imlygic®, BioVex Inc., a subsidiary of Amgen Inc., based in Thousand Oaks, California) has become an option [1]. In refractory cases, ICB is being combined with OVT [2, 3], with off-target or abscopal responses occurring in up to 20% of patients^3^. Although many patients are actualizing the benefits of IO therapies, adverse effects (AEs) of these approaches affect the majority of those treated and new AEs are still emerging. For example, the rapid progression of adult T-cell leukemia-lymphoma (ATLL) following PD-1 inhibitor therapy was recently described [4]. Furthermore, the delivery of IO therapies is complicated by the fact that many of the patients treated in routine clinical practice are excluded from clinical trials due to comorbidities; thus, the efficacy and risks are not effectively attributed to all patients. In particular, the risks and benefits of OVT in patients with concomitant malignancies and/or compromised immune systems are almost completely unknown. Due to concerns for disseminated viral infection, OVT is not recommended in patients with compromised immune systems or with malignancies such as leukemia or lymphoma, though there is little published clinical experience highlighting the risks and benefits of OVT in this population. Currently, the successful administration of T-VEC in a patient on immunosuppressive therapy for an allogeneic heart transplantation^5^ is the only case we are aware of that details OVT delivery to a patient for which it is contraindicated. To date, there have been no reports of the use of dual ICB/OVT in patients with coexistent primary cutaneous ALCL (pcALCL) and metastatic melanoma. In addition, there have been no publications demonstrating Kaposi’s varicelliform eruption after receiving a cycle of T-VEC.

## Case presentation

Herein, we describe an 81-year-old male with concomitant metastatic melanoma and pcALCL whose disease progressed on nivolumab and who then developed Kaposi’s varicelliform eruption following one cycle of T-VEC.

**Fig. 1:**
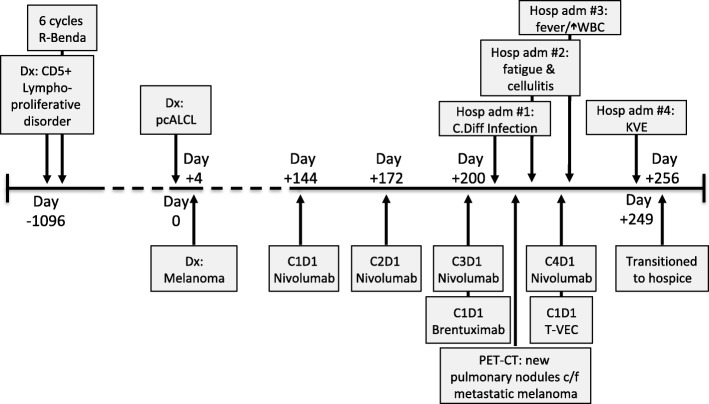
Timeline of the patient’s clinical course. A time reference has been included, with Day 0 referring to the day of the diagnosis of pcALCL. C1D1, cycle 1 day 1; c/f, concerning for; Dx:, diagnosis; Gy, gray; Hosp adm, hospital admission; KVE, kaposi’s varicelliform eruption; pcALCL, primary cutaneous anaplastic large cell lymphoma; R-Benda, rituximab-bendamustine; T-VEC, Talimogene laherparepvec; WBC, white blood cell count; WLE, wide-local excision; XRT, radiotherapy

**Fig. 2:**
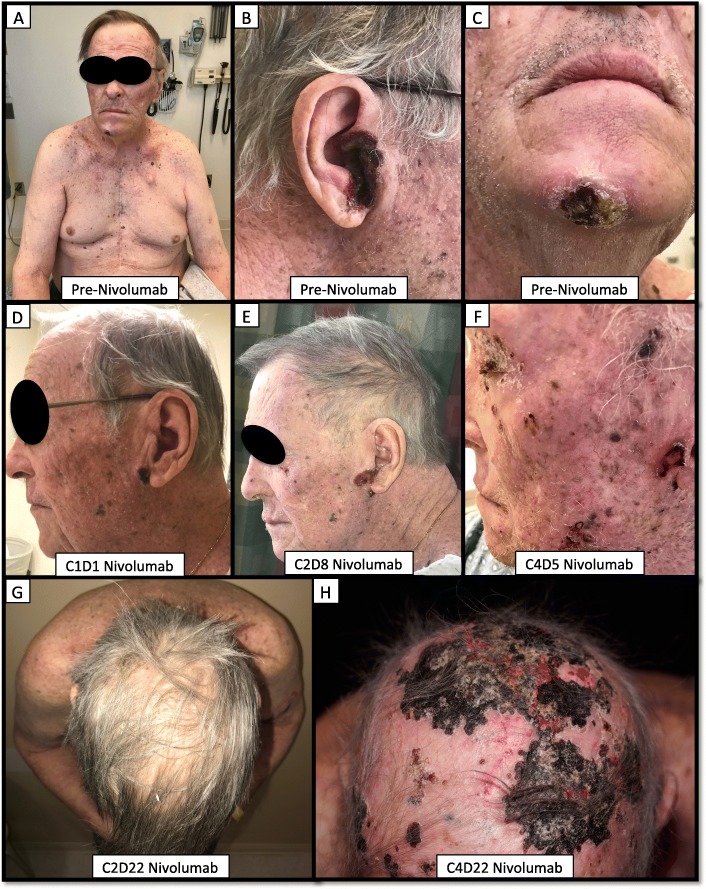
Progression of the patient’s primary cutaneous anaplastic large cell lymphoma. The patient’s lesions prior to nivolumab treatment (**a**-**c**); on treatment (**d**-**h**), with corresponding cycle days denoted in the white box on the bottom of each insert

The patient had a complicated past medical history including coronary artery disease, treated with a coronary artery bypass graft, cerebral vascular accident following a left knee arthroplasty with residual partial aphasia, and a low-grade CD5+ B-cell lymphoproliferative disease, presenting as a large pleural effusion, which was put in a complete remission following 6 cycles of bendamustine and rituximab (R-Benda) (Figure 1). Nearly two and a half years following completion of R-Benda, the patient developed ulcerative plaques on his chin, scalp, lip, right inner canthus and penile foreskin (Figure 2A-C). A skin biopsy was obtained and was consistent with an anaplastic lymphoma kinase (ALK)-negative, CD4^+^ CD30^+^, PD-1^−^, primary cutaneous anaplastic large cell lymphoma (ALCL). In addition to highlighting the cutaneous plaques of ALCL, a staging positron emission tomography-computed tomography (PET-CT) scan demonstrated a 2.7 × 1.5 cm fludeoxyglucose (FDG) avid right axillary lymph node. A core needle biopsy of the lymph node demonstrated melanoma. Five of 10 lymph nodes were positive for melanoma upon a right axillary lymphadenectomy.

A subsequent dermatological evaluation revealed a new 1 cm red/bluish nodule on the right forearm. Surgical excision of the lesion confirmed a 4.7 mm thick, nodular, BRAF wild-type melanoma. Concurrent with the diagnostic work up for his melanoma, the patient underwent targeted radiotherapy to the cutaneous ALCL lesions with excellent response. Following excisions of the right forearm and right axillary melanoma, a re-staging PET-CT demonstrated no additional areas concerning for metastatic melanoma.

The patient was then started on nivolumab for his resected, but high-risk, melanoma. At the time of nivolumab initiation, the patient had pink ulcerative, but improving, plaques of ALCL on his right tragus, chin, left preauricular skin and penile foreskin. He also had faint scaly plaques, without ulceration of the bilateral peri-ocular skin. On cycle 2 day 8 of nivolumab he developed new ulcerative plaques on his upper lip, left upper arm and left pre-auricular skin (Figure 2E) and worsening scale and erythema of his face that were consistent with progressing ALCL. Two new lesions consistent with melanoma recurrence were also noted on the right wrist at the site of the previous melanoma excision. Due to apparent worsening of his ALCL on nivolumab, the decision was made to treat concurrently with radiotherapy and brentuximab. Given the early time point, the appearance of new melanoma nodules were not considered to be a nivolumab failure, and he was continued on therapy.

On cycle 3 day 1 of nivolumab, the patient was given an initial dose of brentuximab vedotin. He also received external beam radiotherapy to his upper lip, left ear and left upper arm. On cycle 3 day 11, the patient was admitted for clostridium difficile-toxin positive colitis, which improved with oral vancomycin. A restaging PET-CT during cycle 3 demonstrated multiple new pulmonary nodules concerning for metastatic melanoma. On cycle 3 day 20, the patient was admitted again secondary to worsening fatigue and a concern for cellulitis surrounding a radiotherapy-treated ALCL lesion of his left upper arm. The patient was treated with antibiotics and improved. During this admission, new ulcerative plaques were noted on the scalp concerning for progression of the ALCL and cutaneous lesions of metastatic melanoma were identified on the right arm and right chest.

Due to worsening disease and declining performance status, the patient’s treatment options were limited. Ipilimumab was deemed inappropriate due to the recent clostridium difficile infection and worsening fatigue. There was also concern that his pcALCL was being exacerbated by ICB and that treatment with brentuximab may have accelerated the melanoma. Thus, after discussing the risks and benefits, the decision was made to discontinue brentuximab and treat the in-transit lesions of melanoma with talimogene laherparepvec concurrently with nivolumab. On cycle 4 day 1 of nivolumab, the patient was treated with 1.7 mL of 1 million plaque-forming-units/mL to three melanoma lesions on the right forearm and two on the right chest. At that visit, the patient was noted to have worsening erythema and scale of his upper extremities, upper chest and face. This was thought to be multifactorial, with asteatosis cutis a prominent feature, as well as either an ICB-related dermatitis or worsening pcALCL. On cycle 4 day 3, the patient presented to clinic febrile (temperature 103.0 F), fatigued and was noted to have a leukocytosis (21,200 white blood cells per microliter). He was subsequently hospitalized for 4 days and the presentation was attributed to an AE of T-VEC. During the admission, the patient had a skin biopsy of the worsening erythema and scale on his face (Figure 2F), which demonstrated features consistent with pcALCL.

**Fig. 3:**
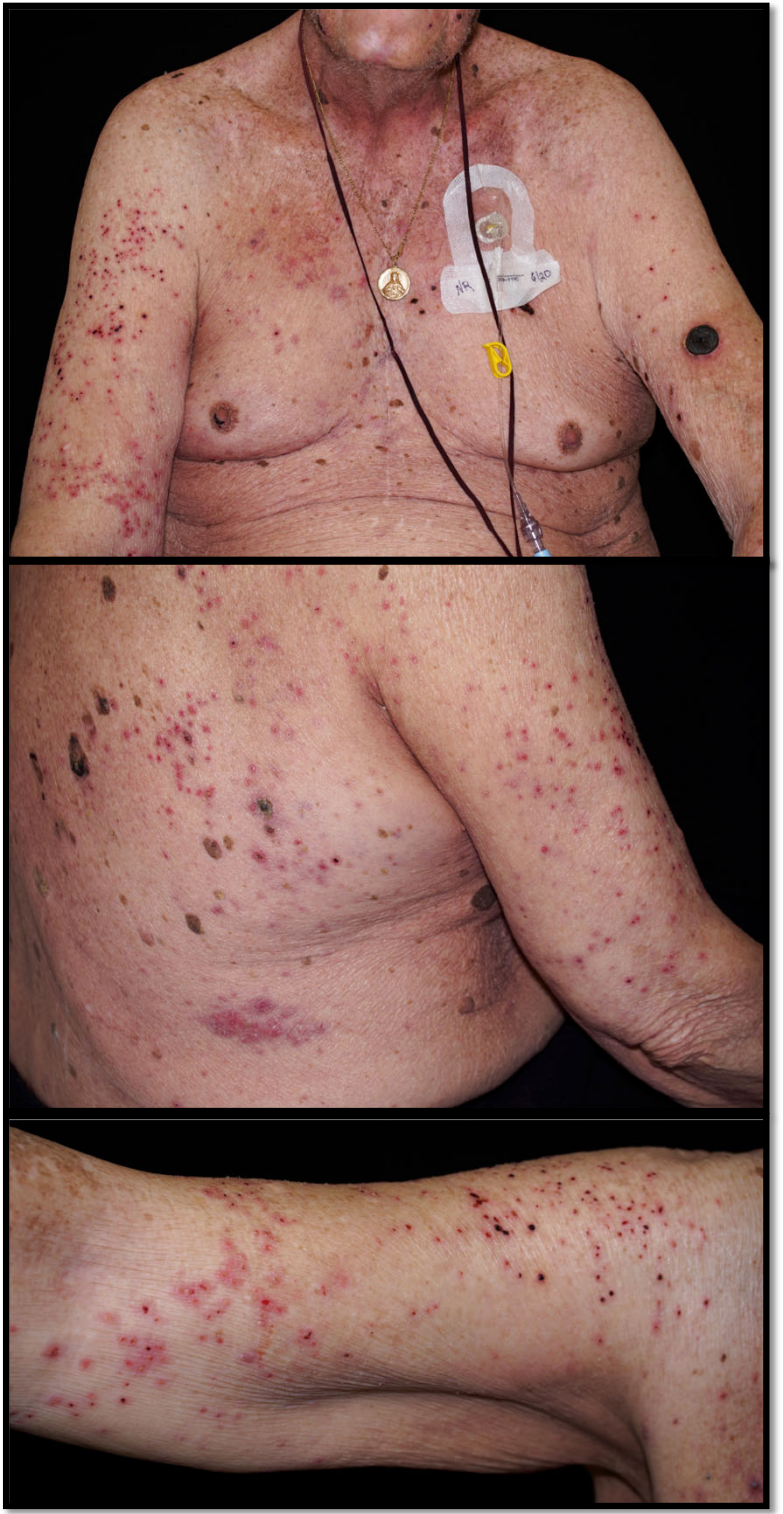
Kaposi’s varicelliform eruption. Scattered 2–3 mm eroded and crusted papules on the bilateral upper arms and trunk. Lesions are concentrated on the right upper arm and trunk

**Fig. 4:**
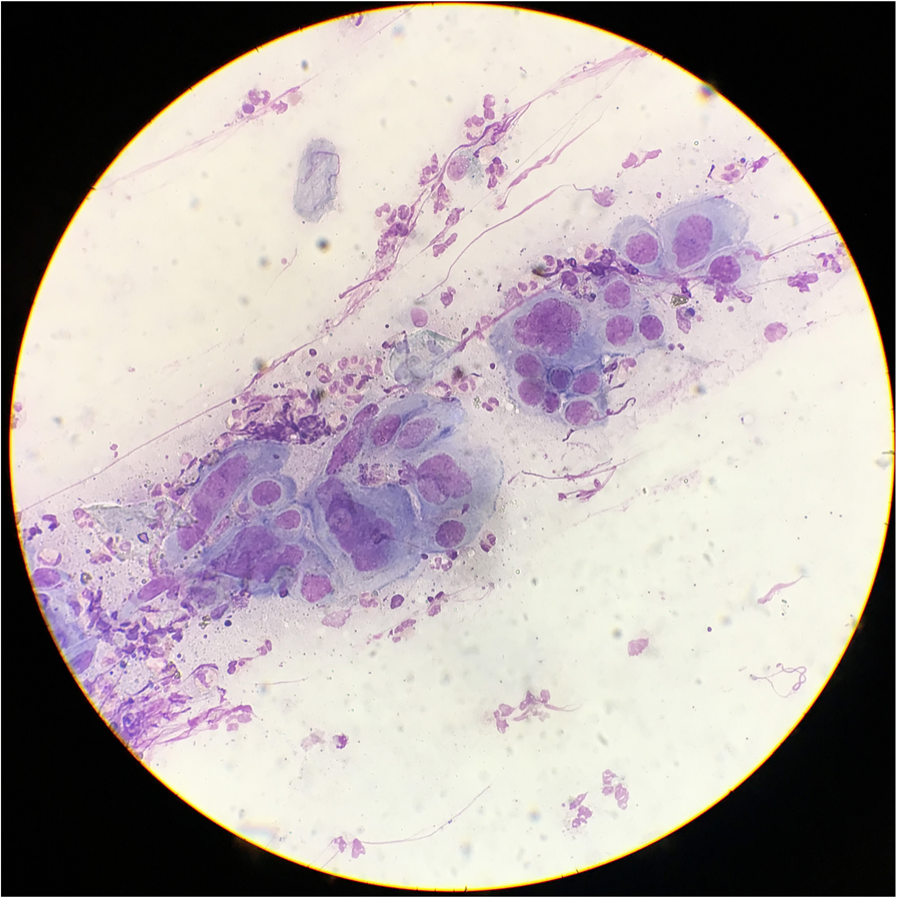
Tzanck smear from a vesicle on the right arm. Under medium power, multinucleated giant cells with viral cytopathic effects, including nuclear molding, are seen

In anticipation of a second cycle of T-VEC, the patient returned to clinic on cycle 4 day 22. On examination, a diffuse eruption of eroded papules was noted on his bilateral upper extremities, chest, flank and back (Figure 3A-C). A few intact vesicles were visible on the right forearm. The lesions were notably asymmetrical in distribution, with the highest density occurring on the right upper arm and right chest. The patient was afebrile and reported mild pruritus of the eruption, which he reported began a few days previously. A Tzanck smear was performed from one of the intact vesicles. Multinucleated giant cells with cytopathic changes were noted (Figure 4). Due to concern for Kaposi’s varicelliform eruption (KVE), the patient was started on intravenous (IV) acyclovir. A direct fluorescence antibody test performed on a vesicle confirmed HSV1 infection. A skin biopsy demonstrated epidermal ulceration with acute inflammation and viral cytopathic effects. HSV I/II-specific immunoperoxidase stain was positive, while a specific immunostain for VZV was negative. HSV viremia was not detected by polymerase chain reaction. He was given 48 hours of IV acyclovir and when the vesicles had completely crusted over, he was discharged on a 14-day course of oral valacyclovir. He experienced a complete resolution of his KVE; however, due to his progressing melanoma, ALCL and declining performance status, the patient was transitioned to hospice care.

## Discussion and concluding remarks

There are several unique aspects to this case. To our knowledge, this is the first reported use of ICB in a patient with both metastatic melanoma and pcALCL. It is also the first report of a patient receiving OVT in the setting of lymphoma. And finally, this is the first publication of Kaposi’s varicelliform eruption developing in the setting of T-VEC.

This case highlights the challenges of treating two concurrent malignancies in the context of immunotherapy. Although the benefits of ICB for melanoma [5–7] and B-cell lymphoma [8, 9] have been well documented, the data for ICB in T-cell lymphomas are more limited, and conflicting results have been reported. Previously, Lesokhin et al. reported 4 of 23 patients with various forms of T-cell lymphoma exhibited responses to nivolumab [10]. In addition, two complete responses have been published in nivolumab-treated patients with ALK-positive systemic ALCL [11, 12]. However recently, Ratner et al. reported rapid progression of ATLL following PD-1 inhibitor therapy [4]. The effects of anti-PD-1 therapy on ALK-negative pcALCL have not been described. Our patient’s ALK-negative pcALCL significantly worsened after the first cycle of nivolumab, and continued to rapidly progress with subsequent doses (Figure 2 D-H). Previously, it was hypothesized that PD-1 may act as a tumor suppressor for T-cell lymphomas, possibly accounting for the rapid progression seen in nivolumab-treated ATLL patients [4]. Our patient’s pcALCL biopsy was negative for PD-1 by immunohistochemistry prior to treatment, making this mechanism less likely to explain the rapid progression of our patient’s ALCL.

Whether the patient’s worsening pcALCL contributed to the development of a hitherto unreported adverse effect of OVT is unclear. AEs are experienced by nearly all patients treated with T-VEC, with chills, pyrexia and injection-site pain being the most common [1]. Additional cutaneous AEs that have been described include injection-site erythema, cellulitis and chronic granulomatous dermatitis [13]. KVE has not previously been reported.

Kaposi’s varicelliform eruption was first described by Moritz Kaposi in 1887 [14]. KVE is the presentation of widespread cutaneous infection of viral pathogens, most commonly herpes simplex virus. KVE has, however, also been reported in the context of other viral infections such as vaccinia, varicella zoster, and coxsackie virus [15]. Although classically seen in patients with atopic dermatitis, in which the eruption is also called “eczema herpeticum”, KVE can also occur in patients with impaired skin barrier function due to a wide variety of conditions. These include: thermal trauma, ichthyosis vulgaris, pemphigus foliaceus, Darier disease, Hailey–Hailey disease, mycosis fungoides, and Sézary syndrome [16].

KVE most commonly presents as small 2–3 mm, discrete, punched-out erosions with hemorrhagic crusts, rather than intact vesicles, similar to what was observed in our patient. Prior to the use of anti-viral therapy, KVE had an approximate mortality rate of 10–50% [17]. Causes of death include viral pneumonia, adrenal hemorrhage, encephalitis, meningitis and bacterial super-infection. Additional causes of morbidity include viral keratitis. Management of KVE typically involves assessment of systemic involvement, ophthalmologic evaluation for keratitis, systemic antiviral therapy and treatment of bacterial superinfection, as necessary.

There is very little data regarding the use of OVT in patients with compromised immunity. As most patients with additional primary malignancies or immunocompromise are excluded from clinical trials, there is little data to guide clinical care in these complex scenarios. T-VEC use in immunosuppressed patients or those with leukemia/lymphoma is not recommend by the product label given the posited risk of disseminated viral infection. However, in certain clinical contexts, when treatment options are limited, the unknown risks of postulated AEs must be weighed heavily with the potential of clinical benefit. Recently, T-VEC was shown to be safe and effective in an allogeneic heart-transplant recipient on cyclosporine [18]. Thus, case reports can provide real-world examples outside of clinical trials or in scenarios cautioned on product labels. Furthermore, it is particularly relevant given that T-VEC is being investigated in clinical trials for patients with refractory cutaneous T-cell lymphoma (e.g. NCT02978625).

We suspect that our patient’s impaired skin barrier secondary to a combination of asteatosis cutis, ICB-mediated dermatitis and worsening cutaneous lymphoma contributed to epidermal spread of the engineered herpes virus, analogous to what is seen in eczema herpeticum, eczema vaccinatum or eczema coxsackium. Epidermal spread, rather than hematological transmission, is supported by a negative HSV1 PCR from the blood and the fact that the highest density of herpetic lesions were concentrated around the sites previously injected with T-VEC: the right arm and right chest. Thus, caution should be used in employing OVT in areas of compromised skin integrity to prevent the development of an eczema herpeticum-like eruption termed “eczema TVECium” or “lymphoma tvecium”. This case also highlights that although engineering efforts were made to attenuate off-target AEs and induce preferential replication within tumors cells-e.g. by deleting the HSV1 virulence genes ICP34.5 and ICP47– [19] T-VEC retains the potential to replicate in non-neoplastic keratinocytes.

Fortunately, our patient had no evidence of keratitis or systemic organ involvement, and his KVE resolved without sequelae. Thus, despite the eruption being widespread, our patient’s KVE was effectively managed with antiviral therapy and the overall clinical impact was limited. Unfortunately, neither ICB nor OVT were effective in controlling the patient’s malignancies and the decision was made to transition to symptom-directed therapy.

In summary, we present a case of a patient with melanoma and pcALCL who was treated with ICB and oncolytic viral therapy and developed a novel adverse effect: eczema TVECium.

## Abbreviations

AE: Adverse effect
ALK: Anaplastic lymphoma kinase
ATLLL: Adult T-cell leukemia-lymphoma
c/f: Concerning for
C1D1: Cycle 1 day 1
DFA: Direct fluorescence antibody
Dx: Diagnosis
FDG: Fludeoxyglucose
Gy: Gray
Hosp adm: Hospital admission
HSV: Herpes simplex virus
ICB: Immune checkpoint blockade
IO: Immunoncology
IV: Intravenous
KVE: Kaposi’s varicelliform eruption
OVT: Oncolytic viral therapy
pcALCL: Primary cutaneous anaplastic large cell lymphoma
PD-1: Programmed-death 1
PET-CT: Positron emission tomography-computed tomography
R-Benda: Rituximab-bendamustine
T-VEC: Talimogene laherparepvec
WBC: White blood cell count
XRT: Radiotherapy

## References

[1] Andtbacka RH, Kaufman HL, Collichio F (2015). Talimogene Laherparepvec improves durable response rate in patients with advanced melanoma. J Clin Oncol.

[2] Puzanov I, Milhem MM, Minor D (2016). Talimogene Laherparepvec in combination with Ipilimumab in previously untreated, Unresectable stage IIIB-IV melanoma. J Clin Oncol.

[3] Sun L, Funchain P, Song JM (2018). Talimogene Laherparepvec combined with anti-PD-1 based immunotherapy for unresectable stage III-IV melanoma: a case series. J Immunother Cancer.

[4] Ratner L, Waldmann TA, Janakiram M, Brammer JE (2018). Rapid progression of adult T-cell leukemia-lymphoma after PD-1 inhibitor therapy. N Engl J Med.

[5] Weber J, Mandala M, Del Vecchio M, et al. Adjuvant Nivolumab versus Ipilimumab in resected stage III or IV melanoma. N Engl J Med. 2017. 10.1056/NEJMoa1709030 28891423

[6] Hodi FS, O'Day SJ, McDermott DF (2010). Improved survival with ipilimumab in patients with metastatic melanoma. N Engl J Med.

[7] Robert C, Long GV, Brady B (2015). Nivolumab in previously untreated melanoma without BRAF mutation. N Engl J Med.

[8] Ansell SM, Lesokhin AM, Borrello I (2015). PD-1 blockade with nivolumab in relapsed or refractory Hodgkin's lymphoma. N Engl J Med.

[9] Chen R, Zinzani PL, Fanale MA (2017). Phase II study of the efficacy and safety of Pembrolizumab for relapsed/refractory classic Hodgkin lymphoma. J Clin Oncol.

[10] Lesokhin AM, Ansell SM, Armand P (2016). Nivolumab in patients with relapsed or refractory hematologic malignancy: preliminary results of a phase Ib study. J Clin Oncol.

[11] Hebart H, Lang P, Woessmann W (2016). Nivolumab for refractory anaplastic large cell lymphoma: a case report. Ann Intern Med.

[12] Rigaud Charlotte, Abbou Samuel, Minard-Colin Véronique, Geoerger Birgit, Scoazec Jean Yves, Vassal Gilles, Jaff Nabaz, Heuberger Laurence, Valteau-Couanet Dominique, Brugieres Laurence (2017). Efficacy of nivolumab in a patient with systemic refractory ALK+ anaplastic large cell lymphoma. Pediatric Blood & Cancer.

[13] Everett AS, Pavlidakey PG, Contreras CM (2018). Chronic granulomatous dermatitis induced by talimogene laherparepvec therapy of melanoma metastases. J Cutan Pathol.

[14] Kaposi M. Pathologie und Therapie der Hautkrankheiten. *Vorlesungen für praktische Aerzte und Studirende.* Vol 3. Vienna: Urban und Schwarzenberg; 1887.

[15] Mathes EF, Oza V, Frieden IJ (2013). “Eczema coxsackium” and unusual cutaneous findings in an enterovirus outbreak. Pediatrics.

[16] Wollenberg A, Wetzel S, Burgdorf WH, Haas J (2003). Viral infections in atopic dermatitis: pathogenic aspects and clinical management. J Allergy Clin Immunol.

[17] Wheeler CE, Abele DC (1966). Eczema herpeticum, primary and recurrent. Arch Dermatol.

[18] Schvartsman G, Perez K, Flynn JE, Myers JN, Tawbi H (2017). Safe and effective administration of T-VEC in a patient with heart transplantation and recurrent locally advanced melanoma. J Immunother Cancer..

[19] Rehman H, Silk AW, Kane MP, Kaufman HL (2016). Into the clinic: Talimogene laherparepvec (T-VEC), a first-in-class intratumoral oncolytic viral therapy. J Immunother Cancer..

